# Left atrial appendage occlusion: an alternative to anticoagulation in atrial fibrillation

**DOI:** 10.3389/fcvm.2026.1889918

**Published:** 2026-10-15

**Authors:** Yosra Messaoudi, Olivier Roux, Pierre Monney, Olivier Muller, Fabian Nietlispach

**Affiliations:** 1 Faculté de Médecine de Sousse, Université de Sousse, Sousse, Tunisia; 2 Department of Cardiology, Ibn El Jazzar Hospital, Kairouan, Tunisia; 3 University Heart Center, University Hospital Zurich, Zurich, Switzerland; 4 Department of Cardiology, Lausanne University Hospital, Lausanne, Switzerland; 5 Department of Cardiology, University of Lausanne, Lausanne, Switzerland

**Keywords:** atrial fibrillation, left atrial appendage occlusion, oral anticoagulation, stroke, systemic embolism

## Abstract

Atrial fibrillation (AF) is the most common sustained arrhythmia. Global Burden of Disease estimates indicate that 52.6 million individuals were living with AF or atrial flutter in 2021, and one in three to one in five persons are expected to develop AF during their lifetime. AF is associated with an increased risk of stroke, systemic thromboembolism, and death. Oral anticoagulation (OAC) is the first-line strategy for preventing AF-associated embolism. However, embolic events still occur in 1%–4% of anticoagulated patients per year, major bleeding occurs in 2%–4% per year, and long-term adherence to direct oral anticoagulants (DOAC) is incomplete. Left atrial appendage occlusion (LAAO) was developed as a mechanical alternative to address these unmet needs. The evidence base has changed substantially over the last 2 years: three randomized controlled trials comparing LAAO with DOAC—OPTION, CHAMPION-AF, and CLOSURE-AF—have now reported. In this update, we summarize the pathophysiological rationale, the technical aspects of the procedure, and the totality of the randomized and observational evidence, including a 2026 meta-analysis of 5,890 randomized patients. Taken together, these data indicate that LAAO reduces non-procedure-related bleeding but does not decrease all-cause major bleeding, intracranial haemorrhage, or mortality, and may carry a higher risk of ischaemic stroke than DOAC. LAAO remains an established option for patients with contraindications to or intolerance of long-term OAC. In DOAC-eligible patients, it has become a randomized-tested alternative, but current evidence supports an individualized, shared decision rather than a universal offer. We outline the remaining controversies—patient selection, device-related thrombus, peri-device leak, post-procedural antithrombotic therapy, and the interpretation of observational data—and the trials designed to resolve them.

## 1 Introduction

Atrial fibrillation (AF) is the most common sustained arrhythmia. The Global Burden of Disease study estimated 52.6 million prevalent cases of AF and atrial flutter worldwide in 2021, with 4.5 million incident cases and 0.34 million deaths; prevalent cases have more than doubled since 1990 (1). One in three to one in five persons are expected to develop AF during their lifetime (2). AF is associated with an increased risk of ischaemic stroke, systemic thromboembolism, and death. AF may also cause coronary embolism: in a consecutive series of 1,776 patients with acute myocardial infarction, coronary embolism accounted for 2.9% of cases, with AF identified as the most frequent underlying cause in 73% of embolic infarctions (3).

Oral anticoagulation (OAC) is the first-line prevention for AF-associated embolism. Although effective in preventing stroke, embolism still occurs in 1%–4% of anticoagulated AF patients per year (4), and there is a significant incidence of major bleeding in these patients (2%–4% per year) (5). Adherence and persistence are additional challenges. A systematic review and meta-analysis of 48 observational studies involving 594,784 patients with AF found that the pooled proportion of patients persisting with direct oral anticoagulants (DOAC) therapy was 69%, while only 66% achieved good adherence (proportion of days covered ≥80%). Non-adherence was associated with an increased risk of stroke (hazard ratio 1.39, 95% CI 1.06–1.81) (6). Overall, a substantial proportion of patients at risk of stroke do not receive or take anticoagulation because of bleeding, contraindications, or patient preference (7).

Left atrial appendage occlusion (LAAO) is an appealing alternative to OAC for patients with non-valvular AF, addressing several unmet needs. LAAO has rapidly gained acceptance in clinical practice, as evidenced by large national registries: the National Cardiovascular Data Registry (NCDR) LAAO Registry enrolled over 38,000 patients in its first 3 years (8), and the SURPASS analysis of the same registry reported on more than 66,000 recipients of a second-generation device (9).

Given the current evidence, should every patient with AF be informed about the available options for stroke prevention to obtain meaningful informed consent for either therapy? This review addresses this question by presenting the evidence, identifying gaps in knowledge, providing an overview of clinical studies, and discussing implications for clinical practice and future research. Because this field is rapidly evolving and occasionally contested, we have deliberately separated the evidence from our clinical opinions; statements reflecting the latter are explicitly introduced by the words “in our view.”

## 2 Thrombogenesis and embolism in atrial fibrillation

The pathogenesis of thrombogenesis in AF is multifactorial and not solely related to stasis in a poorly contractile left atrium. Several mechanisms have been identified in experimental animal models and humans, leading to the recent concept of atrial myopathy (10).

Atrial myopathy combines abnormal atrial contractility (i.e., AF itself) with endothelial dysfunction, inflammation, reduced nitric oxide, activation of growth factors, and stimulation of the renin-angiotensin-aldosterone system. These factors result in atrial remodelling, stasis, and a prothrombotic state (Figure 1) (11).

**Figure 1:**
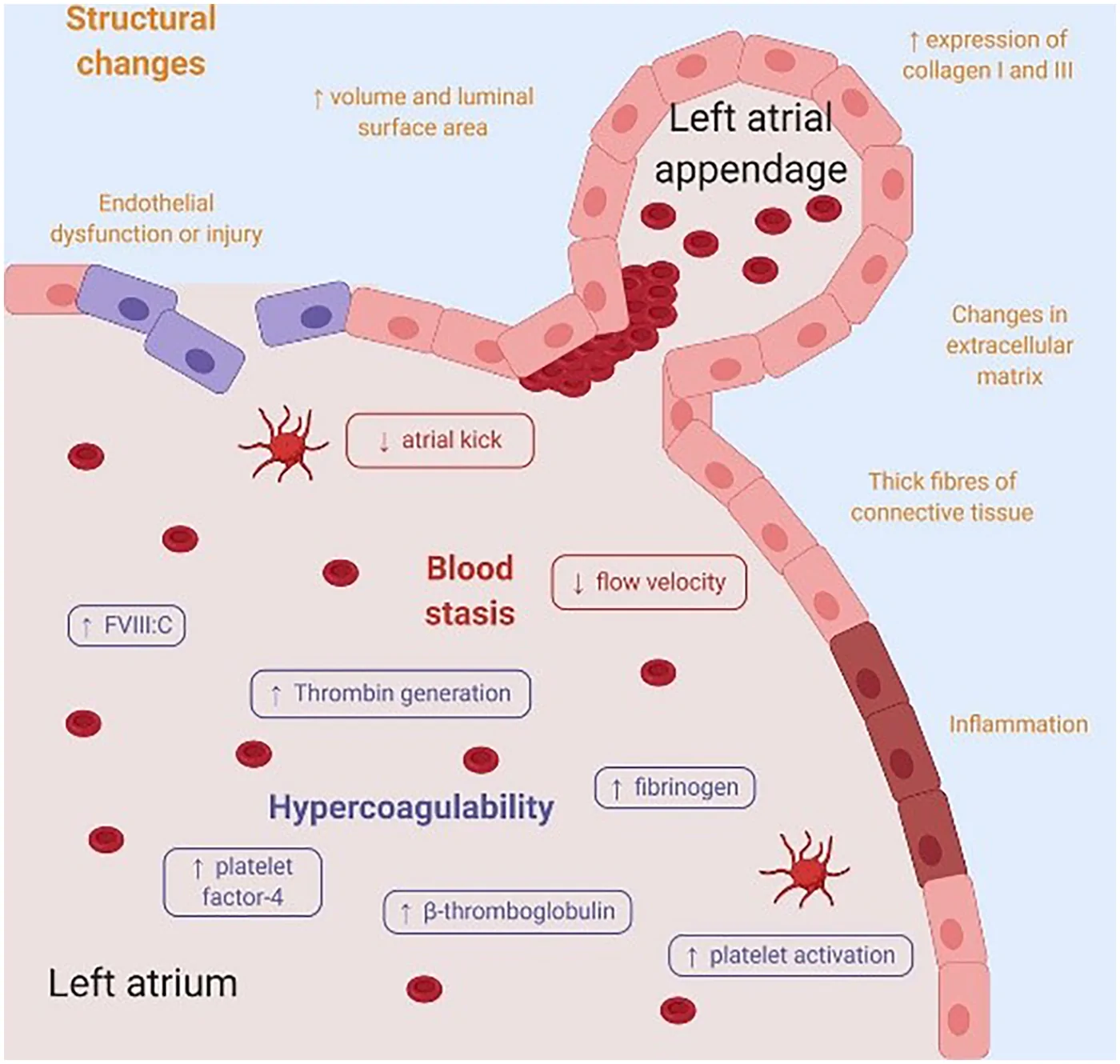
Effects of atrial fibrillation on the various elements of Virchow’s triad (hypercoagulability, structural changes, and blood stasis) (11).

### 2.1 The left atrial appendage and stroke risk

The left atrial appendage (LAA) is a finger-like, trabeculated structure located at the upper anterior wall of the left atrium. It was early identified as the primary site of thrombus formation in AF (12), due to significant stasis and a neurohormonal environment that promotes thrombogenesis and hypercoagulability. Numerous studies have shown that thrombosis outside the LAA occurs rarely in non-valvular AF, and when present, an LAA thrombus is usually concomitant (13–16).

It has been proposed that LAA morphology provides additional predictive value for thrombus formation. A large autopsy study examined the impact of the number of LAA lobes, revealing that the LAA consists of two lobes in half of the population and three lobes in a third of individuals (17). The authors found a correlation between the number of lobes and the presence of LAA thrombus, with most patients having three or more lobes. Furthermore, the LAA has been classified into four morphologies based on its radiographic appearance: cactus, chicken-wing, cauliflower, and windsock (Figure 2) (18). The non-chicken-wing morphology is most closely associated with thrombus formation and embolic stroke, possibly due to a lower LAA peak-flow velocity.

**Figure 2:**
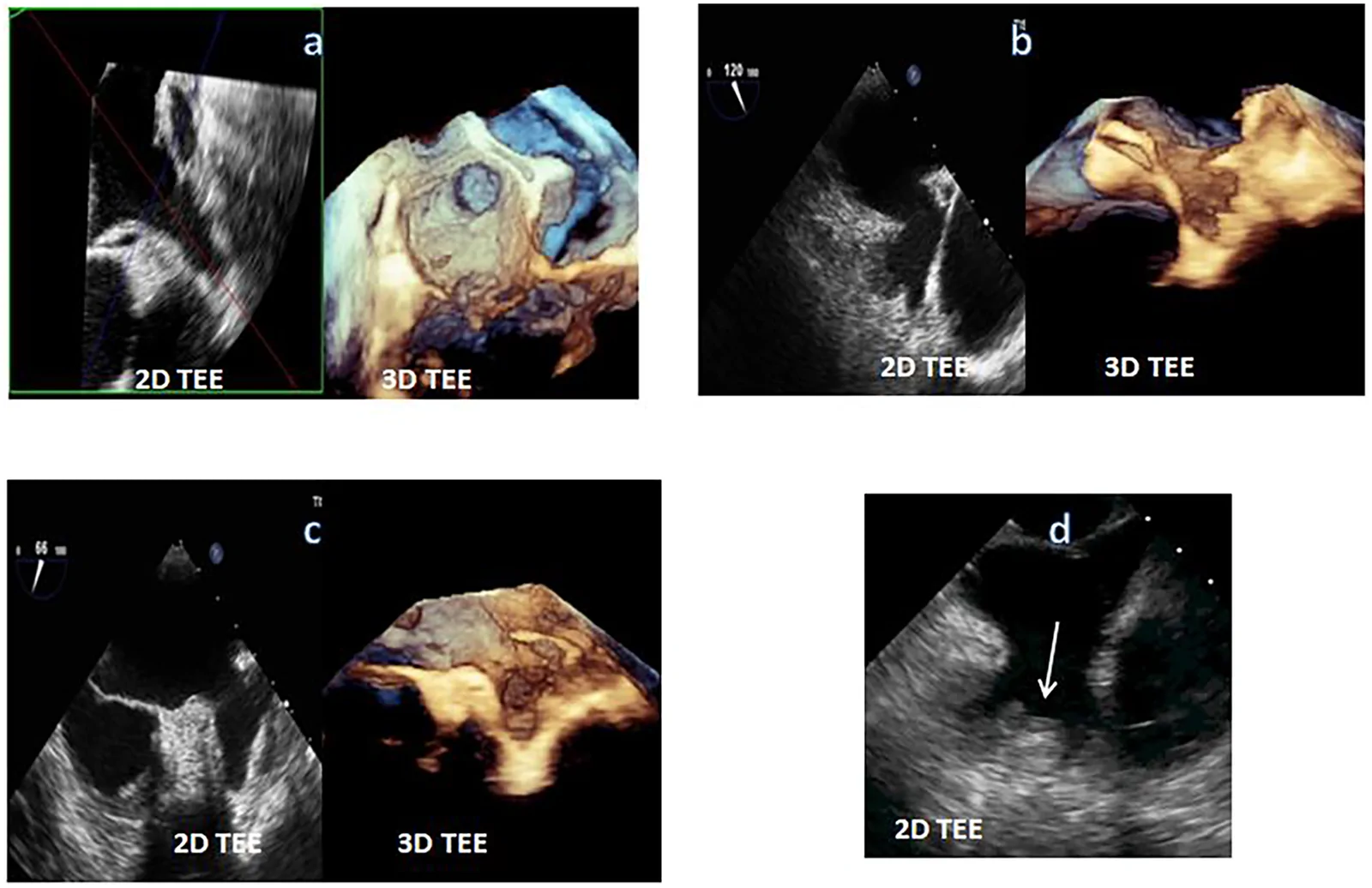
Left atrial appendage morphologies as shown by two- and three-dimensional transoesophageal echocardiography: windsock **(a)**, cactus **(b)**, chicken-wing **(c)**, and cauliflower **(d)** with a thrombus (arrow).

An important mechanistic caveat must be noted. OAC acts systemically, while LAAO acts locally: mechanical exclusion of the appendage cannot protect against thromboembolism originating outside the LAA or against non-cardioembolic stroke mechanisms, such as large-artery atherosclerosis and small-vessel disease, which are common in this elderly, vascular population. In the Stroke Prevention in Atrial Fibrillation trials, 52% of ischaemic strokes in patients with AF were classified as probably cardioembolic, 24% as non-cardioembolic, and 24% as of uncertain cause; among patients receiving warfarin, 56% of breakthrough strokes were non-cardioembolic, compared with 16% of those occurring on aspirin (19). This residual non-appendage risk is an expected limitation of any appendage-directed therapy and should be communicated to patients: while LAAO removes the dominant source of cardioembolism, it does not eliminate all sources of stroke.

In valvular AF (patients with a mechanical prosthetic valve or moderate-to-severe mitral stenosis), left atrial flow is significantly reduced, and the prevalence of left atrial thrombi is up to 45% in both autopsy and echocardiographic studies (20, 21). These patients have not been included in LAAO trials and remain an indication for vitamin K antagonists.

### 2.2 Stroke and thromboembolism prevention in AF: current guidelines

Stroke prevention relies on appropriate risk stratification. Both European and American guidelines recommend using the CHA2DS2-VA(Sc) score alongside an assessment of modifiable bleeding risk factors. OAC is recommended for stroke prevention in patients with elevated thromboembolic risk (22, 23). Over the last decade, DOACs have been shown to be superior to vitamin K antagonists in non-valvular AF regarding safety and efficacy (24). The four available DOACs are the factor Xa inhibitors apixaban, rivaroxaban, and edoxaban, and the direct thrombin inhibitor dabigatran.

Although OAC is recommended as standard care, its implementation can be challenging. Bleeding remains a significant issue even with DOACs, as indicated by annual major bleeding rates of 2%–3.6% in randomized trials (24). Other important challenges include under- and overdosing (especially in underweight patients and those with liver or renal failure) and drug adherence (6).

With respect to LAAO, the 2024 ESC guidelines recommend considering percutaneous LAAO for patients with AF who have contraindications for long-term anticoagulant treatment (Class IIb, Level B). Surgical LAAO may be considered for patients undergoing cardiac surgery with a high stroke risk and contraindications to OAC (Class IIb, Level C) (22). The 2023 ACC/AHA/ACCP/HRS guideline is somewhat more permissive, stating that for patients with AF, a moderate-to-high stroke risk, and a contraindication to long-term OAC because of a non-reversible cause, percutaneous LAAO is reasonable (Class 2a). This represents an upgrade from the previous Class 2b recommendation, justified by accumulating safety and efficacy data (23).

Non-pharmacological alternatives to OAC include surgical exclusion of the LAA—via excision, suture line, stapler, or clip (25)—and transcatheter LAAO (26). Patients with a formal contraindication to anticoagulation, the group currently targeted for percutaneous LAAO by the guidelines, make up approximately 5% of the AF population (27). Dedicated evidence for this group is limited: The ASAP study, often cited in this context, was a prospective, multicentre, non-randomized feasibility study of 150 patients treated with dual antiplatelet therapy after Watchman implantation, not a randomized controlled trial (28). CLOSURE-AF is the first adequately sized randomized trial to directly address this population (29).

It is important to recognize the context in which these recommendations were made. At that time, the pivotal Watchman trials compared a first-generation device with warfarin in OAC-eligible patients, PRAGUE-17 was the only randomized comparison with DOAC, and no adequately powered randomized trial had compared LAAO with DOAC in a broad DOAC-eligible population. Therefore, the Class IIb/2a recommendation is best understood as a reflection of the available evidence when the documents were drafted, rather than a failure to acknowledge existing data; the writing committees include members of the cardiology community. Whether these recommendations should now be revised in light of OPTION, CHAMPION-AF, and CLOSURE-AF is discussed in Section 8.8.

## 3 Percutaneous left atrial appendage occlusion: technical aspects

### 3.1 Current LAAO devices

The first percutaneous LAAO was performed in 2001 using the PLAATO device (Appriva Medical, Sunnyvale, CA, USA), which has not been commercially available since 2006 (30). Before dedicated devices became available, LAAO was performed with non-dedicated devices by pioneers such as Prof. Bernhard Meier in Switzerland.

Current dedicated devices for LAAO are based on three different principles (26):

- Plug principle: endovascular delivery of a lobe obstructing the neck of the LAA, preventing blood flow into the body of the LAA. LAA exclusion relies on endothelialization and sealing of the device. Examples include the Watchman FLX and Watchman FLX Pro (Boston Scientific, Marlborough, MA, USA) and WaveCrest (Biosense Webster, Diamond Bar, CA, USA).
- Pacifier principle: endovascular delivery of a device with a lobe and an additional disc to seal the ostium of the LAA, such as the Amplatzer Cardiac Plug (ACP) and Amulet (both Abbott, Santa Clara, CA, USA), Ultraseal (Cardia Inc., Eagan, MN, USA), and LAmbre (Lifetech, Shenzhen, China).
- Ligation: ligation of the neck of the LAA using a combined endocardial and epicardial approach with the LARIAT device (SentreHEART Inc., Redwood City, CA, USA).

The most commonly used devices in Switzerland, as in most of Europe, are the Watchman and Amplatzer families.

The first-generation Watchman device (Figure 3) was the second dedicated LAAO device after PLAATO and the first studied in randomized trials. It features a self-expanding nitinol frame with a polyethylene terephthalate fabric cap covering about half of the proximal portion; distal fixation anchors secure the device within the LAA trabeculae. Available sizes are 21, 24, 27, 30, and 33 mm, corresponding to the width of the unconstrained device at its proximal shoulders. The device received Conformité Européenne (CE) mark approval in 2005 and Food and Drug Administration (FDA) approval in 2015 for patients with non-valvular AF at high risk of stroke (31).

**Figure 3:**
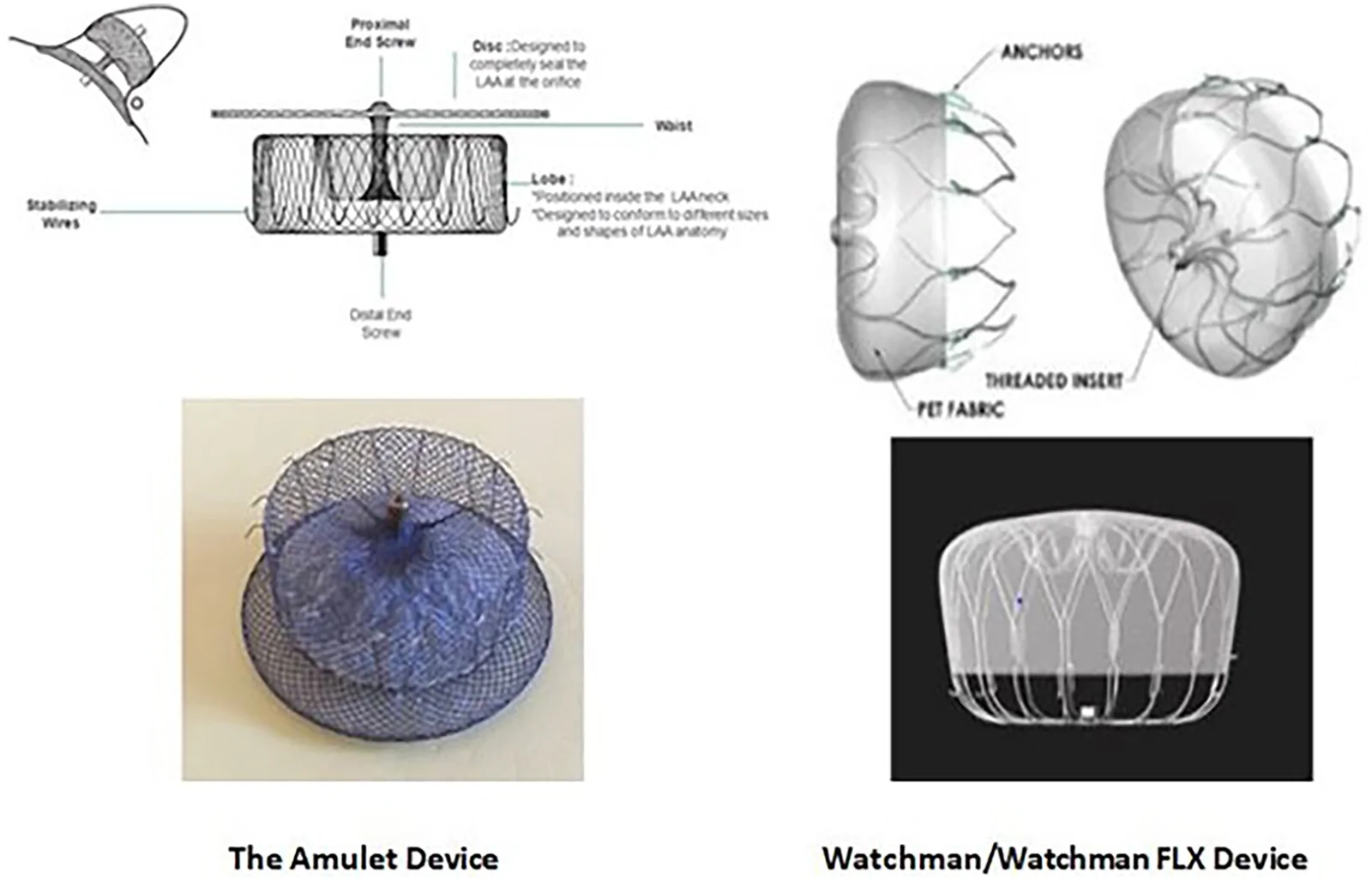
Design of the amulet and Watchman devices.

The Watchman FLX is fully covered by the polyethylene terephthalate membrane, has a closed, atraumatic distal end, and features an increased number of open frame cells. It is available in five sizes (20, 24, 27, 31, and 35 mm) for treating ostia from 15 to 32 mm (32). A significant design change is that, unlike the first-generation device—which could only be partially recaptured—the Watchman FLX can be fully recaptured and repositioned before release. This contributes to higher implant success rates and fewer peri-device leaks with contemporary Watchman iterations compared to those used in pivotal trials: a comparative analysis of the NCDR LAAO Registry showed that the Watchman FLX was associated with higher rates of successful implantation and complete sealing than the first-generation Watchman (33). The Watchman FLX Pro, approved by the FDA in September 2023, adds a thromboresistant polymer coating designed to accelerate endothelialization and reduce device-related thrombus, along with radiopaque visualization markers and an additional 40 mm size, thereby extending the treatable anatomical range (34).

The Amplatzer Cardiac Plug is a self-expanding nitinol mesh featuring a distal lobe and a proximal disc with polyester fabric, connected by a waist. The device is shorter in length than its diameter, allowing it to fit into short appendages. The disc seals the LAA ostium on the left atrial side, while the lobe is implanted into the LAA body. Eight sizes are available based on lobe dimensions, ranging from 16 to 30 mm. The Amulet, a latest-generation device in this family, follows a similar design and is available in eight sizes from 16 to 34 mm, accommodating a broader range of LAA anatomies. Device sizing is based on individual anatomical considerations, and stable implantation requires minimal device compression through oversizing. The Amplatzer occluders received CE mark in 2008 and FDA approval in 2021.

A balanced, evidence-based comparison of the two contemporary platforms is provided in Table 3 and discussed in Section 6.3.

### 3.2 Device implantation procedure

Percutaneous LAAO can be safely performed under local anaesthesia with fluoroscopic guidance or under general anaesthesia with transoesophageal echocardiography (TEE). TEE guidance is also possible without general anaesthesia but carries a higher aspiration risk and may be uncomfortable for the patient. Some operators use intracardiac echocardiography, which allows the procedure to be done under local anaesthesia. While echocardiographic guidance is not mandatory, some operators prefer it for performing the transseptal puncture (Table 1).

**Table 1:** Imaging strategies for device implantation: comparison of different methods.

Imaging strategy	Advantages	Disadvantages
Fluoroscopy only	Patient-friendly and resource-friendly; safe; faster recognition of complications (e.g., pain in case of acute pericardial effusion); no general anaesthesia	No second imaging modality; requires the ability to perform a fluoroscopy-guided transseptal puncture; may require more contrast agent
Fluoroscopy and transoesophageal echocardiography	Second imaging modality; more precise selection of the transseptal puncture site; direct visualization of the landing zone	Morbidity of TEE (bleeding, perforation); usually requires general anaesthesia; longer recovery; more resources; longer procedure (risk of thrombus formation)
Fluoroscopy and intracardiac echocardiography	Second imaging modality without the need for general anaesthesia; less radiation exposure for the echocardiographer	Expensive; second femoral venous puncture with a higher risk of vascular complications; two-dimensional images only with most systems; additional catheter in the atrium not guided by a wire; potential distraction of the operator; longer procedure time

Vascular access is achieved via a femoral vein, and a transseptal puncture is performed using standard techniques, ideally targeting inferior and posterior in the fossa ovalis. Although a patent foramen ovale (PFO) is located superiorly and anteriorly, successful LAAO is achievable in over 95% of patients when the PFO is used for left atrial access; thus, the PFO should be used when present (35). If the LAA cannot be accessed easily via the PFO, one should not insist on this approach but instead perform a dedicated transseptal puncture. The same applies to atrial septal defects.

Pre-procedural imaging should be conducted using either computed tomography or TEE to rule out a pre-existing LAA thrombus. Interestingly, even when no thrombus is detected on TEE or computed tomography before the procedure, small thrombi are often present in the very distal, trabeculated part of the LAA; these do not hinder safe LAAO. In patients who have been uninterruptedly anticoagulated before the procedure, it may be acceptable to omit pre-procedural imaging. In any case, we recommend performing angiographic imaging of the LAA once the left atrium has been accessed to detect potentially relevant thrombi.

Intraprocedural imaging of the LAA is mandatory, and we strongly recommend acquiring angiographic images even when echocardiography is available. The LAA is depicted in right anterior oblique caudal and cranial projections. Only then can the operator decide where the device can be safely implanted—a decision that often does not align with the standard echocardiographic “landing zone.” Angiographic sizing uses the outer diameter of the sheath as a reference (e.g., 5.4 mm for a 14 F Amulet sheath). Oversizing the landing zone by approximately 20% is recommended. As the LAA is distended by injections, operators do not need to adjust for left atrial pressure, contrary to some publications.

Once the device has been delivered, a long, continuous tug test should be performed while monitoring for any dislocation. Angiographically, it is important to obtain images in a perfectly perpendicular view to the device and to inject vigorously into the LAA to confirm contact of the lobe at both the upper and lower parts of the LAA from at least two different angles. In those angles, the lobe should also appear compressed (tyre-shaped or strawberry-shaped). Only then can the device be safely detached from the delivery cable.

Procedure duration depends on operator experience and the imaging strategy. In our practice, using a fluoroscopy-guided approach in experienced hands, the procedure typically takes about 30 min. Published real-world data provide a more generalizable estimate: in the 3,822 consecutive procedures performed in the United States immediately after FDA approval—71% by operators who had not participated in the clinical trials—implantation was successful in 95.6% of cases with a median procedure time of 50 min (range 10–210 min), a pericardial tamponade rate of 1.02%, a procedure-related stroke rate of 0.078%, and a device embolization rate of 0.24% (36).

## 4 Procedural and clinical outcomes: LAAO device trials and studies

LAAO has now been compared with oral anticoagulation or with physician-directed best medical care in six randomized controlled trials, and with another occluder in one further randomized trial (Amulet IDE); all seven trials are summarized in Table 2. LAAO has also been compared with OAC in several propensity-score-matched observational studies. Two features of this evidence base must be kept in mind: first, the comparator differed across trials (warfarin, DOAC, or physician-directed best medical care); second, and more importantly, the populations differed significantly, meaning each trial only supports conclusions for the population it enrolled.

**Table 2:** Randomized controlled trials comparing left atrial appendage occlusion with oral anticoagulation or another device.

Trial (year)	Population enrolled	*N*	Comparator	Primary endpoint	Main result	Principal limitation
PROTECT-AF (2009/2014)	Warfarin-eligible non-valvular AF	707	Warfarin	Stroke, cardiovascular death, systemic embolism	Non-inferior at 18 months; met superiority at extended follow-up, driven by haemorrhagic stroke and cardiovascular death	Procedure-related complications 8.7%; warfarin comparator; first-generation device
PREVAIL (2014)	OAC-eligible non-valvular AF	407	Warfarin	Two efficacy co-primary endpoints and a safety endpoint	Procedural safety markedly improved (4.2% vs. 8.7%); one of two efficacy co-primary endpoints not met	Underpowered; warfarin comparator; first-generation device
PRAGUE-17 (2020; 4-year 2022)	Prior cardioembolism and/or clinically relevant bleeding; CHA2DS2-VASc ≥3 and HAS-BLED ≥2	402	DOAC (95% apixaban)	Composite of ischaemic, bleeding, and procedural events	Non-inferior at 20 months and at 4 years; non-procedural bleeding significantly reduced	Modest sample size; selected high-risk population; not applicable to the general DOAC-eligible population
Amulet IDE (2021; 5-year 2025)	Non-valvular AF at high stroke risk, suitable for LAAO	1,878	Watchman 2.5 (device vs. device)	Safety, effectiveness, and LAA occlusion	Non-inferior; at 5 years ischaemic stroke/systemic embolism 7.4% vs. 7.1%, major bleeding 20.1% vs. 20.0%, death 28.7% vs. 31.1% (all NS); fewer device factors preceding events with Amulet	Comparator is the first-generation Watchman; higher periprocedural complication rate with Amulet
OPTION (2025)	AF with elevated stroke risk undergoing catheter ablation	1,600	Oral anticoagulation	Safety: non-procedure-related bleeding. Efficacy: death, stroke, or systemic embolism	Superior for bleeding (8.5% vs. 18.1%; *p* < 0.001); non-inferior for efficacy (5.3% vs. 5.8%)	Applies only to patients undergoing ablation
CHAMPION-AF (2026)	DOAC-eligible non-valvular AF; mean age 72 years, mean CHA2DS2-VASc 3.5	3,000	DOAC	Cardiovascular death, stroke, or systemic embolism at 3 years	Non-inferior (5.7% vs. 4.8%); non-procedure-related bleeding 10.9% vs. 19.0% (*p* < 0.001); ischaemic stroke/systemic embolism 3.2% vs. 2.2%; all ISTH major bleeding 5.9% vs. 6.4%; device-related thrombus 4.8%	Event rates lower than projected, making the 4.8 percentage-point margin permissive; industry-funded
CLOSURE-AF (2026)	AF at high risk of stroke and bleeding or with contraindication to OAC; mean age 77.9 years, mean CHA2DS2-VASc 5.2	912	Physician-directed best medical care (mostly DOAC)	Stroke, systemic embolism, major bleeding, or cardiovascular/unexplained death	Non-inferiority NOT met: 16.8 vs. 13.3 events per 100 patient-years; adjusted HR 1.28 (95% CI 1.01–1.62)	Enrolment from 2018 with earlier device generations; not powered for individual components

### 4.1 Randomized comparisons with warfarin

The Watchman device was evaluated in two large prospective randomized trials, PROTECT-AF (37, 38) and PREVAIL (39), in which it was compared with chronic warfarin therapy in patients with non-valvular AF.

PROTECT-AF was a non-inferiority trial. Between 2005 and 2008, investigators enrolled 707 warfarin-eligible patients with non-valvular AF and randomized them to either warfarin or the Watchman device; the device group received warfarin for 45 days after implantation. The primary efficacy endpoint of stroke, cardiovascular death, and systemic embolism showed no difference between the two groups. At extended follow-up, the device met criteria for both non-inferiority and superiority for the composite endpoint, largely due to a reduction in haemorrhagic stroke and cardiovascular death (38). However, there was a high rate of procedure-related complications (8.7%), primarily serious pericardial effusion (4.8%). Because of these safety concerns, the FDA mandated a further confirmatory randomized trial. PREVAIL, which included 407 OAC-eligible patients, demonstrated a significant reduction in procedural complications compared with PROTECT-AF, attributed to operator experience (4.2% vs. 8.7%; *p* = 0.004) (39).

The long-term incidence of severe bleeding was examined in the 1,114 patients enrolled in PROTECT-AF and PREVAIL, showing that LAAO significantly reduces bleeding beyond the procedural period, particularly after OAC has been discontinued (40).

In our view, these two trials provide the strongest evidence in favour of LAAO over vitamin K antagonists. However, they should not be extrapolated to a comparison with DOACs: warfarin carries a substantially higher risk of intracranial haemorrhage than DOACs (24), so the benefit observed with LAAO in these trials may not persist with contemporary comparators.

### 4.2 Randomized comparisons with direct oral anticoagulants

PRAGUE-17 was a randomized non-inferiority trial comparing LAAO (Watchman, Watchman FLX, or Amulet) with DOAC (95% apixaban) in 402 patients with non-valvular AF and a history of cardioembolism, clinically relevant bleeding, or both, with a CHA2DS2-VASc score ≥3 and a HAS-BLED score ≥2. The primary endpoint, a composite of ischaemic, bleeding, and procedural events, was non-inferior for LAAO compared with DOAC, and this result was maintained after 4 years of follow-up; non-procedural bleeding was significantly reduced with LAAO (41, 42). PRAGUE-17 was, however, a modestly sized trial enrolling a high-risk population selected for previous bleeding or cardioembolism and does not address the broader DOAC-eligible population.

The OPTION trial randomized 1,600 patients with AF and elevated stroke risk who were undergoing catheter ablation to LAAO with the Watchman FLX (performed concomitantly in 40.8% and sequentially in 59.2%) or to continued oral anticoagulation. At 36 months, LAAO was superior for the primary safety endpoint of non-procedure-related major or clinically relevant non-major bleeding (8.5% vs. 18.1%; *p* < 0.001) and non-inferior for the primary efficacy endpoint of death, stroke, or systemic embolism (5.3% vs. 5.8%; *p* < 0.001 for non-inferiority) (43). OPTION, therefore, establishes LAAO as a reasonable alternative to continued anticoagulation specifically in patients undergoing AF ablation.

CHAMPION-AF is the largest randomized trial to date and the first to compare LAAO with DOAC in a broad population of patients suitable for long-term anticoagulation. A total of 3,000 patients (mean age 72 years, mean CHA2DS2-VASc score 3.5) were randomized to LAAO with the Watchman FLX or to DOAC. At 3 years, the primary composite endpoint of cardiovascular death, stroke, or systemic embolism occurred in 5.7% of the device group and 4.8% of the anticoagulation group (difference 0.9 percentage points, 95% CI −0.8 to 2.6; *p* < 0.001 for non-inferiority against a pre-specified margin of 4.8 percentage points). Non-procedure-related bleeding, the primary safety endpoint, was significantly lower with LAAO (10.9% vs. 19.0%; hazard ratio 0.55, 95% CI 0.45–0.67; *p* < 0.001 for superiority) (44).

The results of CHAMPION-AF are more nuanced than the non-inferiority headline suggests, and these nuances are important to this review. First, the observed event rates were considerably lower than projected, making the absolute non-inferiority margin of 4.8 percentage points comparatively permissive. Second, ischaemic stroke or systemic embolism was numerically more frequent in the device arm (3.2% vs. 2.2%), while cardiovascular death (2.7% in both arms), haemorrhagic stroke (0.4% in both arms), and all-cause mortality (5.0% vs. 4.9%) were essentially identical. Third, the bleeding advantage was driven by non-procedure-related events: when including procedure-related bleeding, ISTH major bleeding rates were similar in both arms (5.9% vs. 6.4%). Fourth, device-related thrombus was detected on imaging in 4.8% of device recipients and considered clinically relevant in 1.8%. The accompanying editorial concluded that these findings justify offering LAAO to selected DOAC-eligible patients within a shared decision, but do not establish the device as an equivalent substitute for anticoagulation (45).

CLOSURE-AF addressed the population in which LAAO is most often performed in contemporary practice. In this investigator-initiated trial conducted at 42 German sites, 912 patients with AF at high risk of both stroke and bleeding or with contraindications to long-term anticoagulation (mean age 77.9 years, mean CHA2DS2-VASc score 5.2, mean HAS-BLED score 3.0) were randomized to LAAO or to physician-directed best medical care, which included DOAC when tolerated. After a median follow-up of 3 years, the primary composite endpoint of stroke, systemic embolism, major bleeding, or cardiovascular or unexplained death occurred at 16.8 per 100 patient-years in the device group vs. 13.3 per 100 patient-years in the medical-therapy group; LAAO did not meet the pre-specified criterion for non-inferiority (adjusted hazard ratio 1.28, 95% CI 1.01–1.62) (29).

In our view, CLOSURE-AF should not be interpreted as a negative trial invalidating LAAO as a therapeutic option. The individual components of the composite endpoint occurred at similar rates in both groups, and the trial was not powered for those endpoints. Enrolment began in 2018, when first-generation devices and less experienced operators were still common, and the majority of the control group received DOAC, making the comparison against modern medical therapy rather than no therapy. However, the trial requires careful consideration of the value of a procedural intervention in a frail, older, and highly comorbid population, where the upfront procedural risks and early bleeding may offset benefits that accumulate over the years. The accompanying editorial raised the broader question of whether LAAO is being used more widely than the evidence supports (46). Patient selection—specifically the recognition that expected survival must be sufficient for a procedural investment to be worthwhile—emerges as a central determinant of benefit.

### 4.3 Meta-analysis of the randomized evidence

The four randomized trials comparing LAAO with DOAC (PRAGUE-17, OPTION, CHAMPION-AF, and CLOSURE-AF) were pooled in a 2026 meta-analysis including 5,890 patients, with 2,949 assigned to LAAO and 2,941 to DOAC, and a median follow-up of 36–42 months (47). The findings are as follows: There was no significant difference in all strokes, haemorrhagic strokes, systemic embolism, all-cause mortality, or cardiovascular death. LAAO was associated with a numerically higher risk of ischaemic stroke (relative risk 1.36, 95% CI 0.99–1.89; *p* = 0.06), which became statistically significant after excluding the OPTION trial (relative risk 1.45, 95% CI 1.03–2.06; *p* = 0.03). LAAO significantly reduced non-procedure-related major bleeding (relative risk 0.79, 95% CI 0.65–0.97) and non-major bleeding (relative risk 0.47, 95% CI 0.39–0.57), whereas all major bleeding (relative risk 0.97, 95% CI 0.81–1.17) and intracranial haemorrhage (relative risk 0.76, 95% CI 0.45–1.26) did not differ significantly between strategies.

This pooled analysis is, in our view, the most informative summary currently available, reframing the clinical question. Compared to DOAC, LAAO offers a reduction in non-procedure-related bleeding at the potential cost of an increased risk of ischaemic stroke, with no demonstrated effect on mortality, intracranial haemorrhage, or all-cause major bleeding. The magnitude of these trade-offs varies across patient phenotypes: CHAMPION-AF and OPTION enrolled younger patients at lower risk, while PRAGUE-17 and CLOSURE-AF included older patients with significantly greater thromboembolic and bleeding risks. The evidence does not support viewing LAAO as a universally equivalent alternative to systemic anticoagulation across all AF phenotypes.

### 4.4 Registry data

In addition to the randomized data, a substantial amount of registry data has been published on real-world clinical practice. The Continued Access Protocol registry followed the 502 patients enrolled in the LAAO arm of PROTECT-AF, plus 460 additional non-randomized registry patients, and showed a significant decline in procedure- or device-related adverse events, highlighting the role of operator experience in improving safety (48).

Evaluating Real-Life Clinical Outcomes in Atrial Fibrillation Patients Receiving the Watchman Left Atrial Appendage Closure Technology (EWOLUTION) is a prospective, multicentre registry reporting on 1,021 patients receiving a Watchman device. OAC was contraindicated in 62% of patients, and 45% had a prior stroke. Antiplatelet therapy was used in 83%, while vitamin K antagonists were used in only 8%. Implantation was successful in 98.5%, and procedure-related adverse events occurred in 2.8% of patients within 7 days, a significant reduction compared to PROTECT-AF, PREVAIL, and the Continued Access registry (49).

The NCDR LAAO Registry enrolled over 38,000 patients with a Watchman device implanted in the United States from January 2016 to December 2018. Patients were generally older and had more comorbidities than those in pivotal trials (mean age 76.1 ± 8.1 years; mean CHA2DS2-VASc 4.6 ± 1.5; mean HAS-BLED 3.0 ± 1.1). The registry reported prior clinical bleeding in approximately 70% of patients and intracranial bleeding in approximately 12% (vs. none in the randomized trials), with 7.9% discharged without OAC. Despite this higher-risk population, the implant success rate was high (98%), and major in-hospital adverse events were low (2.2%) (8). The subsequent SURPASS analysis of the same registry, involving over 66,000 recipients of the Watchman FLX, confirmed these findings, showing further reductions in major periprocedural adverse events and high rates of effective seal at 1 year (9).

Registry data are essential for assessing procedural safety in unselected practice and tracking the effects of iterative device improvements. However, they cannot establish comparative efficacy against anticoagulation, as they are uncontrolled and the patients selected for LAAO differ systematically from those maintained on OAC.

### 4.5 Propensity-matched observational comparisons

The first propensity-matched study comparing LAAO with OAC was the APPLY study. In this real-world study, 500 patients receiving LAAO with Amplatzer devices at two Swiss centres (mean age 73.9 years, mean CHA2DS2-VASc 4.3) were compared with a matched cohort of 500 patients treated with vitamin K antagonists or DOAC (mean age 74.1 years, mean CHA2DS2-VASc 4.3). After a mean follow-up of 2.7 ± 1.5 years, LAAO demonstrated a net clinical benefit over medical therapy, with superior efficacy, similar safety, and a reduction in all-cause and cardiovascular mortality (50). Comparable findings have been reported by other groups (51, 52). A smaller Italian single-centre retrospective study compared LAAO with DOAC after 1:1 propensity-score matching (92 patients per group, mean CHA2DS2-VASc 4.3); at 2 years, rates of thromboembolic events (7.3% vs. 6.3%; *p* = 0.77), major bleeding (6.3% vs. 6.3%; *p* = 1.00), and all-cause mortality (10.4% vs. 15.6%; *p* = 0.28) were similar (53).

These observational data must be interpreted with caution, as they significantly qualify claims of a mortality benefit. The mortality advantage of LAAO reported in propensity-matched cohorts has never been reproduced in a randomized trial: In the pooled randomized evidence, all-cause and cardiovascular mortality are equivalent (47). Several biases likely account for this discrepancy. Patients referred for and accepted to undergo elective structural procedures are systematically healthier and expected to live longer than similarly scored patients managed medically—a “healthy-candidate” bias that propensity matching cannot address, as frailty, functional status, cognitive status, and life expectancy are rarely captured in administrative or registry datasets. Conversely, intolerance to anticoagulation is a marker of frailty and adverse prognosis. Therefore, we believe the claim that LAAO confers a mortality benefit over OAC is unsupported; it arises solely from observational comparisons subject to residual confounding and should be reported as such.

### 4.6 Cost-effectiveness

A cost-effectiveness analysis based on pooled 5-year data from PROTECT-AF and PREVAIL modelled LAAO with the Watchman device as cost-effective compared with warfarin and DOAC over a lifetime horizon, becoming cost-saving after several years (54). These are model-based projections derived from trial data, not measured economic outcomes, and their conclusions depend on the assumed comparator, event rates, device durability, time horizon, and health-system costs. They should therefore be described as supporting, rather than demonstrating, economic attractiveness, and they require re-estimation now that randomized comparisons with DOAC are available.

## 5 Antithrombotic therapy after LAAO

Similar to other cardiovascular implants, LAAO devices can develop thrombus on the non-endothelialized free surface, potentially resulting in thromboembolic events. The optimal post-interventional antithrombotic regimen and its duration remain among the least well-resolved aspects of the procedure.

The regimens used in the randomized trials were heterogeneous, and this heterogeneity is informative. In PROTECT-AF and PREVAIL, patients received warfarin plus aspirin for 45 days, followed by dual antiplatelet therapy (DAPT) for 6 months and aspirin indefinitely thereafter (37, 39). In PRAGUE-17, the recommended regimen after LAAO was DAPT for 3 months followed by single antiplatelet therapy, with anticoagulation used in selected patients (41). In OPTION and CHAMPION-AF, both using the Watchman FLX, protocol-directed strategies included a short post-implantation period of anticoagulation or DAPT before transitioning to single antiplatelet therapy (43, 44). No trial of LAAO vs. OAC was designed to compare post-procedural antithrombotic strategies, and therefore none of these regimens can be considered validated by those trials.

Registry data support less intensive regimens. In EWOLUTION, 83% of patients were discharged on antiplatelet therapy alone, while only 8% received a vitamin K antagonist, with low rates of device-related thrombus and thromboembolism (49). A Danish cohort reported favourable outcomes with aspirin alone in patients at high bleeding risk (55), and single antiplatelet therapy has been deemed feasible for patients with anticoagulation contraindications (16). However, these observational data do not establish equivalence with more intensive regimens.

Two randomized trials have directly addressed this question, both favouring short-term anticoagulation over DAPT. In ADALA, low-dose apixaban was compared with DAPT (aspirin plus clopidogrel) after LAAO; the composite of thromboembolic events, device-related thrombus, and major bleeding at 3 months was lower with low-dose apixaban, driven by increased major bleeding and more device-related thrombus in the DAPT arm (56). The larger ANDES trial randomized 510 patients (mean age 77 years) across 13 centres in Canada and Europe to receive either a DOAC or DAPT for 60 days after LAAO. Device-related thrombus at 60 days was not significantly reduced (1.5% vs. 4.1%; *p* = 0.11), but the composite safety outcome of death, stroke, bleeding, or device-related thrombus was significantly lower with DOAC (22.5% vs. 34.9%; *p* = 0.003), primarily due to less bleeding (17.4% vs. 24.9%; *p* = 0.038) (57). However, both trials were powered for surrogate or composite endpoints and did not include a single-antiplatelet or no-therapy arm.

In our view, although no definitive trial exists, the following approach is reasonable. For patients who can tolerate short-term anticoagulation, a DOAC at a reduced dose, where appropriate, for 45 days to 3 months, followed by single antiplatelet therapy, is the best randomized-supported default. For patients with contraindications to anticoagulation, DAPT for 1–3 months or aspirin alone are acceptable alternatives, though the evidence supporting them is observational. In patients who cannot take aspirin, such as those with cerebral amyloid angiopathy, complete discontinuation of antithrombotic therapy after the initial post-implantation period may be considered; however, the supporting evidence is limited and observational, so the decision should be individualized rather than applied universally. In the largest series addressing this question, 431 high-bleeding-risk patients were discharged on single antiplatelet therapy after Amplatzer LAAO, and 173 of them (41.7%) were receiving no antithrombotic therapy at 6 months. Device-related thrombus was detected in 1.5%, and the 1-year rate of ischaemic stroke was 2.2% (95% CI 1.1–4.2), comparable to rates reported with more intensive regimens and with a low rate of major bleeding (58). In patients with a history of intracranial haemorrhage or cerebral amyloid angiopathy, a systematic review of 14 studies involving 1,235 patients concluded that LAAO appears safe and effective but graded the quality of evidence as poor, with the regimens in the included studies being single or dual antiplatelet therapy or short-term anticoagulation, rather than complete cessation (59). No randomized trial has evaluated the withdrawal of all antithrombotic therapy after LAAO. Therefore, we believe this strategy should be reserved for patients for whom all antithrombotic agents pose a prohibitive bleeding risk, following documented exclusion of device-related thrombus and significant peri-device leak on post-implantation imaging. Beyond this initial period, continuation of aspirin should be guided by factors other than AF or LAAO, such as concomitant coronary artery disease.

Whether the choice of device should influence this decision cannot currently be answered. The most frequently cited comparison, the Amulet IDE trial, indicated that device factors preceding stroke or cardiovascular death were less common with the Amulet than with the first-generation Watchman (60), so this finding cannot be applied to current practice. The only randomized comparison that included the contemporary Watchman FLX is SWISS-APERO, where 77% of patients allocated to Watchman received the FLX device. Device-related thrombus at 45 days was numerically less frequent with the Amulet than with the Watchman, both on computed tomography (0.9% vs. 3.0%) and on transoesophageal echocardiography (2.1% vs. 5.5%). However, these differences are based on very few events, the trial was not powered for this endpoint, and the Amulet was associated with a higher rate of procedural complications in the same trial (61). In addition, the thromboresistant coating of the Watchman FLX Pro is specifically designed to reduce device-related thrombus and has never been compared head-to-head with the Amulet. Therefore, no device can be recommended over another solely based on the potential for a shorter antithrombotic regimen, and device selection should be guided by the considerations outlined in Section 6.3.

## 6 Peri-device leak and device-related thrombus

### 6.1 Peri-device leak

Peri-device leaks (PDL) are not uncommon and have been shown to increase the risk of subsequent ischaemic events. According to the FDA, PDL greater than 5 mm are considered significant, and current practice is either to continue OAC or perform a second procedure to close the leak; a PDL of 5 mm or less is generally accepted as sufficient LAA occlusion. However, the clinical consequences of these smaller leaks are not fully characterized. Dukkipati et al. demonstrated that a PDL of 5 mm or less at 1 year after LAAO with the Watchman device was associated with an increased risk of thromboembolism (62). Therefore, all efforts should be made to minimize leaks during implantation, and the ability of contemporary devices to be fully recaptured and repositioned is directly relevant to this goal (32, 33). Case series have shown the feasibility of closing leaks with plugs, occluders, or coils, but long-term data on these approaches remain limited.

### 6.2 Device-related thrombus

Device-related thrombus (DRT) is the second major device-specific hazard. In an international registry of 711 patients from 37 centres, DRT was detected within 45 days in 24.9% of patients, between 45 and 180 days in 38.8%, between 180 and 365 days in 16.0%, and beyond 1 year in 20.3%, indicating that the risk extends beyond the early post-implantation period. DRT was linked to a higher risk of the composite outcome of death, stroke, or systemic embolism (hazard ratio 2.37, 95% CI 1.58–3.56) and specifically of ischaemic stroke (hazard ratio 3.49, 95% CI 1.35–9.00). Independent predictors included hypercoagulability, pericardial effusion, renal insufficiency, implantation depth greater than 10 mm from the pulmonary vein limbus, and non-paroxysmal AF (63). A subsequent consensus-based classification proposed a standardized approach to recognizing and managing DRT (64). In CHAMPION-AF, DRT was detected on imaging in 4.8% of patients receiving a Watchman FLX and was deemed clinically relevant in 1.8% (44). DRT remains a significant, quantifiable limitation of the therapy, and the need for imaging surveillance is a practical consequence.

### 6.3 Comparison between contemporary devices

Device comparisons are often presented selectively, so we have aimed to report the full balance of available evidence; a structured summary is provided in Table 3.

**Table 3:** Balanced comparison of the two contemporary left atrial appendage occlusion platforms.

Characteristic	Watchman FLX/Watchman FLX Pro	Amulet
Occlusion principle	Plug (single lobe)	Pacifier (lobe plus proximal disc)
Available sizes	FLX: 20, 24, 27, 31, and 35 mm (ostia 15–32 mm); FLX Pro adds a 40 mm size	16–34 mm (eight sizes)
Recapture/repositioning	Fully recapturable and repositionable (the first-generation Watchman was only partially recapturable)	Fully recapturable and repositionable
Surface treatment	Full polyethylene terephthalate membrane; FLX Pro adds a thromboresistant polymer coating designed to accelerate endothelialization	Polyester patches on lobe and disc
Peri-device leak	Substantially reduced compared with the first-generation device; higher residual leak than Amulet on computed tomography in SWISS-APERO	Lower rate of peri-device leak than Watchman FLX on computed tomography (SWISS-APERO)
Device-related thrombus	4.8% detected on imaging in CHAMPION-AF (1.8% clinically relevant); 3.0% at 45 days on computed tomography in SWISS-APERO. The FLX Pro thromboresistant coating is intended to reduce this risk but has not been compared head-to-head with the Amulet	Fewer device factors (thrombus or leak ≥3 mm) preceding stroke or cardiovascular death than the first-generation Watchman 2.5 in Amulet IDE; 0.9% at 45 days on computed tomography in SWISS-APERO (1 vs. 3 events; the trial was not powered for this endpoint)
Pericardial effusion	Reference device; lower risk	Significantly higher risk than Watchman in a network meta-analysis of six RCTs (RR 2.12, 95% CI 1.45–3.09) and more frequent late effusions in the MAUDE database
Rare serious complications	No pulmonary artery injury reported in the MAUDE analysis	12 pulmonary artery injuries with five deaths reported in the MAUDE analysis (voluntary reporting, no denominator)
Randomized clinical outcomes	No significant difference in stroke or mortality between devices (Amulet IDE at 5 years; network meta-analysis; SWISS-APERO at 3 years)	Same
Randomized evidence vs. anticoagulation	PROTECT-AF, PREVAIL (warfarin); OPTION, CHAMPION-AF (DOAC)	PRAGUE-17 (mixed devices); CATALYST vs. DOAC ongoing

The Amulet IDE trial randomized 1,878 patients to the Amulet or the first-generation Watchman. At 5 years, rates of ischaemic stroke or systemic embolism (7.4% vs. 7.1%), the composite of stroke, systemic embolism, or cardiovascular death (20.3% vs. 20.7%), major bleeding (20.1% vs. 20.0%), and all-cause mortality (28.7% vs. 31.1%) did not differ significantly. Device factors—DRT or PDL ≥3 mm—preceded stroke or cardiovascular death more frequently in the Watchman group (63 vs. 31 patients), and more Amulet patients were free of oral anticoagulation at 5 years (94.0% vs. 90.9%; *p* = 0.009) (60). These advantages must be weighed against the higher rate of periprocedural complications observed with the Amulet in the same trial, a point we did not previously highlight and that is relevant to device selection.

SWISS-APERO is the only randomized head-to-head comparison of the Amulet with the contemporary Watchman FLX. At 1 year, the Amulet had less peri-device leak on computed tomography, but at the cost of more procedural complications (61, 65). At 3 years, the composite ischaemic endpoint occurred less frequently with the Amulet (18% vs. 31%; hazard ratio 0.58; *p* = 0.06), a difference that did not reach significance in the intention-to-treat analysis and was primarily driven by cardiovascular mortality (66). The trial was not powered for clinical endpoints, and these results should be considered hypothesis-generating.

Two recent analyses challenge claims of superiority for either platform. A network meta-analysis of six randomized trials involving 3,666 patients found no difference between the Amulet and the Watchman regarding stroke (relative risk 1.48, 95% CI 0.64–3.46), mortality (relative risk 1.00, 95% CI 0.59–1.70), thromboembolism, or device embolization, but noted a significantly higher risk of pericardial effusion with the Amulet (relative risk 2.12, 95% CI 1.45–3.09) (67). An analysis of the FDA Manufacturer and User Facility Device Experience (MAUDE) database identified 290 reports of pericardial effusion associated with the Amulet compared to 33 with the Watchman; late effusions accounted for 34% of Amulet reports vs. 6% of Watchman reports, and 12 cases of pulmonary artery injury with five deaths were reported with the Amulet, while none occurred with the Watchman (68). MAUDE is a voluntary, passive reporting system without denominators, making it subject to substantial reporting bias; absolute rates cannot be derived from it, and differences in market share and device generations complicate interpretation. Nevertheless, the trend aligns with the randomized meta-analytic data.

Overall, we believe both contemporary platforms provide comparable protection against stroke and mortality. The Amulet appears to achieve more complete sealing and fewer device-related thrombi, while the Watchman FLX seems to have a lower risk of pericardial effusion and the rare but serious complication of pulmonary artery injury. An additional caveat applies to the comparative literature: most randomized comparisons used the first-generation Watchman 2.5 as the comparator, and the design changes in the Watchman FLX and FLX Pro—such as full recapture, complete membrane coverage, additional sizes, and a thromboresistant coating —are expected to have narrowed or eliminated some of the observed differences (32–34). Therefore, device choice should be guided by anatomy, operator experience, and the individual balance of bleeding and procedural risk, rather than by an assumption of general superiority of either device.

## 7 Follow-up after LAAO

After discharge, we recommend a TEE between months 3 and 6 as a final examination, focusing on thrombus formation and peri-device leak. In patients at high bleeding risk, where antithrombotic therapy is to be shortened, an initial TEE may be performed after 6 weeks to rule out thrombus formation. In patients for whom TEE is not feasible or is refused, the same information can be obtained through contrast-enhanced computed tomography. Given that device-related thrombus may develop late and that a peri-device leak of 5 mm or less is not necessarily benign (62, 63), we consider at least one dedicated imaging study post-implantation to be mandatory, and any new or unexplained neurological events after LAAO should prompt repeat imaging, regardless of the time since implantation.

## 8 Unresolved questions and controversies

### 8.1 Which patients should be offered LAAO?

Each randomized trial supports conclusions only for the enrolled population, which differ substantially. PROTECT-AF and PREVAIL enrolled warfarin-eligible patients and used warfarin as a comparator. PRAGUE-17 enrolled high-risk patients with previous cardioembolism or clinically relevant bleeding. OPTION enrolled patients undergoing AF ablation. CHAMPION-AF enrolled DOAC-eligible patients at moderate risk. CLOSURE-AF enrolled older patients at high risk of both stroke and bleeding and did not demonstrate non-inferiority. Currently, there is no randomized evidence supporting LAAO in patients with low bleeding risk who tolerate DOAC well and have no other reason to avoid anticoagulation. CLOSURE-AF warns against assuming benefits in the frailest patients. We believe the population with the most favourable benefit-risk balance includes those with a contraindication to, intolerance of, or a major complication from long-term anticoagulation, where life expectancy warrants the upfront procedural risk. The resulting stratification, along with the practical decision sequence we propose, is summarized in Figure 4.

**Figure 4:**
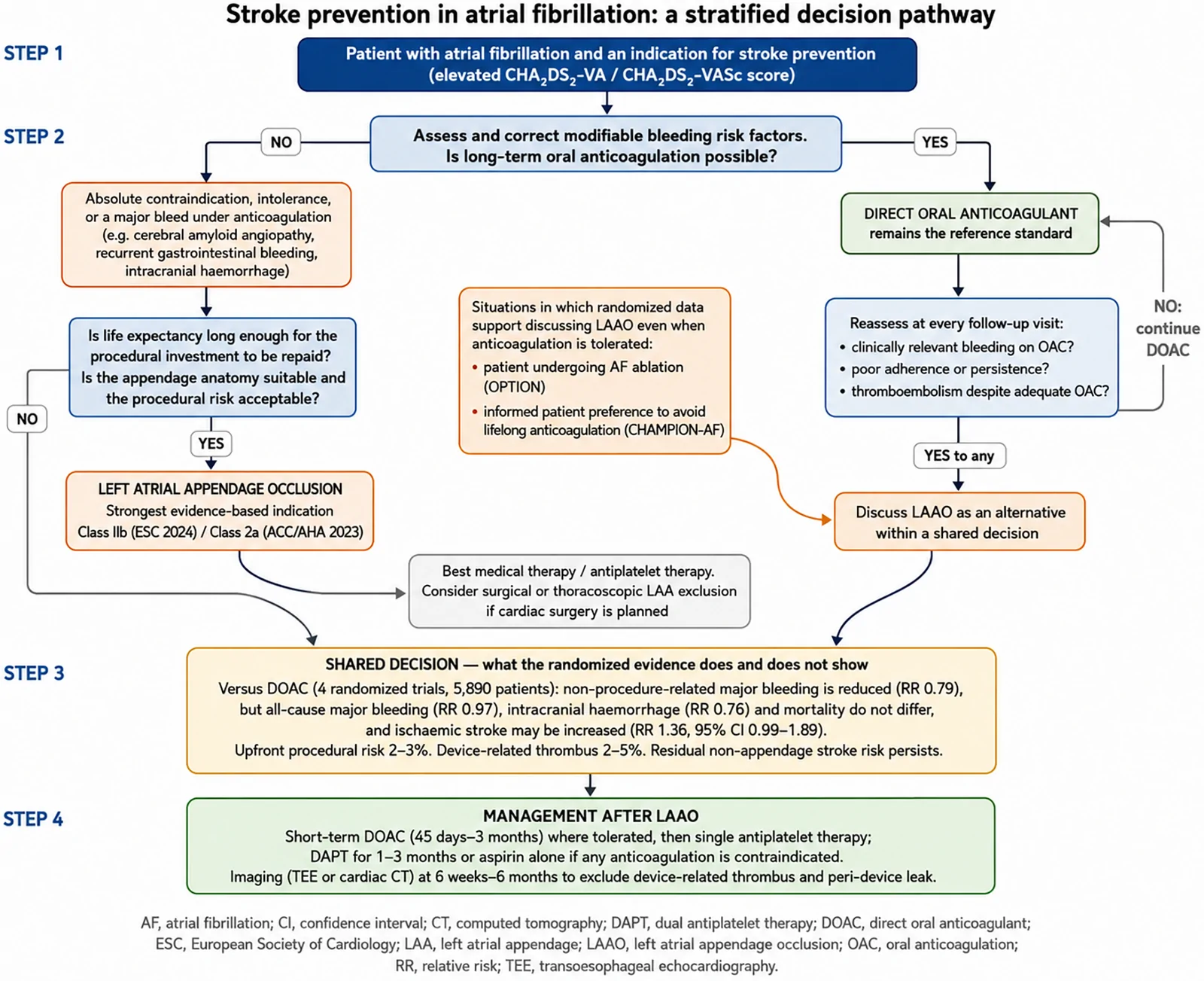
Proposed decision pathway for stroke prevention in atrial fibrillation, incorporating oral anticoagulant eligibility, bleeding risk, and shared decision-making. AF, atrial fibrillation; DOAC, direct oral anticoagulant; LAA, left atrial appendage; LAAO, left atrial appendage occlusion; OAC, oral anticoagulation; SAPT, single antiplatelet therapy.

### 8.2 Is there a residual ischaemic stroke penalty?

The consistent numerical excess of ischaemic stroke with LAAO across randomized trials, which reached statistical significance in the pooled analysis after excluding OPTION (47), is mechanistically plausible: LAAO addresses only appendage-derived thromboembolism, while approximately one-quarter of strokes in patients with AF are non-cardioembolic (19), and device-related thrombus and peri-device leak add device-specific risks (62, 63). Whether this signal represents a true class effect and whether it is mitigated by newer devices and better antithrombotic management remains to be established and requires dedicated, adequately powered evaluation.

### 8.3 Does the bleeding benefit survive the procedural risk?

The reduction in non-procedure-related bleeding with LAAO is both consistent and substantial. However, when procedure-related bleeding is included, all-cause major bleeding was not significantly different from DOAC in either CHAMPION-AF or the pooled randomized analysis, and intracranial haemorrhage—arguably the most feared bleeding event and the one most often cited to justify LAAO—did not differ significantly (44, 47). Whether longer follow-up would eventually reveal a divergence, as the one-off procedural hazard is balanced against an accumulating anticoagulation-related bleeding risk, is a plausible hypothesis but remains unproven.

### 8.4 What is the optimal antithrombotic regimen and its duration?

Randomized evidence now favours short-term anticoagulation over DAPT (56, 57), but no trial has compared short-term anticoagulation with single antiplatelet therapy or no antithrombotic therapy, which is the key comparison for patients whose bleeding risk motivated the procedure. The optimal duration is also unknown, and endothelialization is incomplete in a proportion of patients at conventional follow-up intervals.

### 8.5 How should observational data be weighed?

As discussed in Section 4.5, the mortality benefit of LAAO reported in propensity-matched cohorts is not replicated in randomized trials, most plausibly due to the selection of healthier candidates for elective procedures. We believe that observational and randomized evidence should not be presented interchangeably, and survival claims should be limited to what randomized data support—which, at present, is equivalence (47).

### 8.6 Which device, and does device generation matter?

As outlined in Section 6.3, the two contemporary platforms perform similarly for stroke and mortality, though they have different complication profiles. Most comparative data were generated with earlier device generations, and the pace of device improvement currently exceeds the rate at which comparative outcome data can be accumulated. Ongoing head-to-head trials, along with results from the CATALYST trial comparing the Amulet with DOAC, will be necessary before firm conclusions can be drawn.

### 8.7 Surgical and prophylactic LAA closure

Surgical exclusion of the LAA during cardiac surgery is supported by strong randomized evidence in patients with AF. In LAAOS III, 4,811 patients with AF and a CHA2DS2-VASc score of at least two undergoing cardiac surgery for another indication were randomized to concomitant surgical LAA occlusion or no occlusion. Most patients continued anticoagulation, resulting in a reduced risk of ischaemic stroke or systemic embolism (4.8% vs. 7.0%; hazard ratio 0.67, 95% CI 0.53–0.85) without an increase in perioperative complications (69). It is important to emphasize that LAAOS III enrolled only patients with AF, and its results were obtained in addition to, not instead of, anticoagulation.

The question of whether prophylactic LAA exclusion should be performed in patients without AF is entirely separate and lacks randomized evidence. A meta-analysis of randomized and propensity-score studies in cardiac surgery patients without AF suggested a possible reduction in ischaemic stroke, but the included studies were heterogeneous and largely non-randomized (70). The LeAAPS trial, which is randomizing 6,500 patients without pre-existing AF but at increased risk of AF and stroke undergoing cardiac surgery to LAA exclusion or no exclusion, is designed to answer this question (71). Until those results are available, we do not recommend prophylactic LAA exclusion in patients without AF outside of a clinical trial.

### 8.8 Should guideline recommendations be expanded?

The current Class IIb (ESC) and Class 2a (ACC/AHA/ACCP/HRS) recommendations predate OPTION, CHAMPION-AF, and CLOSURE-AF (22, 23). The new randomized evidence is genuinely bidirectional: It strengthens the case for LAAO in DOAC-eligible patients undergoing ablation and, more cautiously, in selected DOAC-eligible patients generally, while simultaneously weakening the case for LAAO in the frail, high-bleeding-risk population where it is most often performed today. Expert consensus documents from professional societies have already suggested broader use of the procedure (26, 72), and contemporary editorial opinions are now correspondingly divided (45, 46). We believe that upgrading the recommendation for patients with contraindications or intolerance to OAC is justified; however, extending it to unselected DOAC-eligible patients is not yet supported and should await the results of CATALYST and longer follow-up of completed trials. Ongoing and recently completed randomized trials are summarized in Table 4.

**Table 4:** Recently completed and ongoing randomized trials of left atrial appendage occlusion.

Trial (identifier)	Question addressed	Intervention/control	Device	*n*	Status and result
CHAMPION-AF (NCT04394546)	LAAO in non-valvular AF eligible for long-term DOAC	LAAO/DOAC	Watchman FLX	3,000	Published 2026. Non-inferior for the primary composite; non-procedure-related bleeding reduced; ischaemic stroke numerically higher
CLOSURE-AF (NCT03463317)	LAAO in AF at high bleeding risk or with contraindication to OAC	LAAO/best medical care	CE-marked devices	912	Published 2026. Non-inferiority NOT met (adjusted HR 1.28, 95% CI 1.01–1.62)
OPTION (NCT03795298)	LAAO in AF patients undergoing catheter ablation	LAAO/oral anticoagulation	Watchman FLX	1,600	Published 2025. Superior for non-procedural bleeding; non-inferior for efficacy
CATALYST (NCT04226547)	LAAO in non-valvular AF eligible for long-term DOAC	LAAO/DOAC	Amulet	2,650	Active, not recruiting; results awaited
STROKECLOSE (NCT02830152)	LAAO after intracerebral haemorrhage	LAAO/medical therapy	Amulet	750	Ongoing
CLEARANCE (NCT04298723)	LAAO in AF with a history of intracranial haemorrhage	LAAO/medical therapy	Watchman FLX	550	Ongoing
COMPARE-LAAO (NCT04676880)	LAAO in AF with contraindication to OAC	LAAO/antiplatelet or no therapy	Watchman FLX or Amulet	609	Ongoing
LeAAPS (NCT05963698)	Prophylactic surgical LAA exclusion in cardiac surgery patients WITHOUT atrial fibrillation	LAA exclusion/no exclusion	AtriClip	6,500	Enrolment completed; results awaited
WATCH-TAVR (NCT03173534)	LAAO in AF patients undergoing TAVR	TAVR + LAAO/TAVR + medical therapy	Watchman	350	Completed
CONFORM (NCT05147792)	Head-to-head device trial	CLAAS device/Watchman FLX or Amulet	CLAAS	1,400	Ongoing
WaveCrest 2 (NCT03302494)	Head-to-head device trial	WaveCrest/Watchman	WaveCrest	1,550	Ongoing

## 9 Conclusion

LAAO is an established alternative to OAC in specific populations: patients with previous major bleeding under anticoagulation, those at high bleeding risk where anticoagulation cannot be maintained (e.g., cerebral amyloid angiopathy or recurrent gastrointestinal bleeding), patients with a true contraindication to OAC, and those with recurrent embolism despite adequate anticoagulation. In patients undergoing AF ablation, OPTION supports LAAO as an alternative to continued anticoagulation (43). In the broader DOAC-eligible population, CHAMPION-AF has shown non-inferiority for the composite of cardiovascular death, stroke, and systemic embolism, along with a reduction in non-procedure-related bleeding (44). However, the pooled randomized evidence shows no mortality benefit, no reduction in all-cause major bleeding or intracranial haemorrhage, and a possible excess of ischaemic stroke (47). Conversely, CLOSURE-AF failed to demonstrate non-inferiority in older, high-risk patients (29).

Given this evidence, we believe LAAO should be discussed as a treatment option for patients in whom anticoagulation is problematic, and patients seeking non-pharmacological alternatives deserve a balanced account of what is and is not known. When informing patients, it is crucial to state that LAAO is a complex procedure with upfront risks: major periprocedural complications occurred in 2.2% of patients in the NCDR registry (8) and in 2.8% in EWOLUTION (49), with a 1.02% rate of tamponade in the United States post-approval experience (36). This risk is reduced but not eliminated by operator experience and contemporary devices (9). The counterpart is a durable reduction in non-procedural bleeding and freedom from long-term anticoagulation—the reason LAAO has been termed a “mechanical vaccination,” providing protection with 100% adherence (73)—a metaphor that remains appealing but should not obscure the residual, non-appendage stroke risk that the procedure cannot address.

Future research should prioritize the identification of phenotypes where the benefit–risk balance is genuinely favourable, particularly concerning frailty and life expectancy; adequately powered assessment of the ischaemic stroke signal; randomized comparisons of post-procedural antithrombotic regimens, including single antiplatelet therapy; the impact of the latest device generations on device-related thrombus and peri-device leaks; and the role of prophylactic LAA exclusion in patients without AF. Until these questions are resolved, patient selection and genuine shared decision-making—not universal offering—should guide the use of this therapy.
